# Phenolic Antioxidants Isolated from the Flowers of *Osmanthus fragrans*

**DOI:** 10.3390/molecules170910724

**Published:** 2012-09-07

**Authors:** Chien-Ya Hung, Yu-Cheng Tsai, Kuo-Yu Li

**Affiliations:** Department of Food Nutrition, Chung Hwa University of Medical Technology, No.89, Wenhua 1st St., Rende Dist., Tainan City 71703, Taiwan; Email: renee012783@yahoo.com.tw (Y.-C.T.); looking19990817@yahoo.com.tw (K.-Y.L.)

**Keywords:** *Osmanthus fragrans*, Oleaceae, antioxidative activity, DPPH scavenging activity, H_2_O_2_ scavenging ability, phenolic antioxidant

## Abstract

*O. fragrans* has slightly less antioxidative activity than green tea. Five phenolic compounds, tyrosyl acetate (**1**), (+)-phillygenin (**2**), (8*E*)-ligustroside (**3**), rutin (**4**), and verbascoside (**5**), were isolated from the CHCl_3_ sub-extract of *O. fragrans*. The structures were elucidated by interpreting their spectral data. Evaluation of the antioxidative property of the isolated (+)-phillygenin (**2**), rutin (**4**), and verbascoside (**5**) revealed strong DPPH radical scavenging activity, with IC_50_ values of 19.1, 10.3, and 6.2 μM, respectively. These isolates also exhibited an H_2_O_2_ scavenging ability, with IC_50_ values of 10.5, 23.4, and 13.4 μM, respectively.

## 1. Introduction

**Figure 1:**
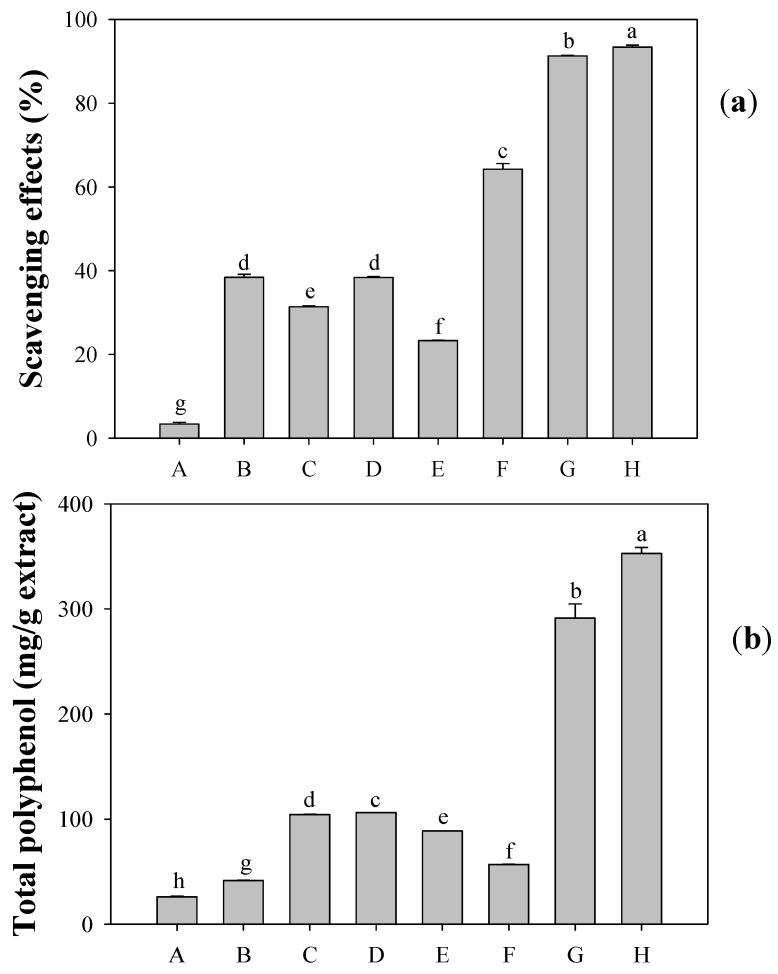
Scavenging effect on DPPH radicals (**a**) and the content of total polyphenols (**b**) of methanol extractof green tea (H) and seven flowers: *G. globosa* (A); *N. nucifera* (B); *H. bracteatum* (C); *C. morifolium* (D); *M. grosvenori* (E); *C. indicum* (F); and *O. fragrans* (G).^a^^–g^ Values in each column with different letters are significantly different (*p* *<* 0.05). Each value is mean ± standard derivation of three replicates.

In China, some flowers are commonly added to tea to increase or improve its taste. The added flowers are Gomphrena globosa, Helichrysum bracteatum, Chrysanthemum morifolium, Momordica grosvenori, Chrysanthemum indicum, Nelumbo nucifer, Osmanthus fragrans, and others. A preliminary antioxidative test was carried out to evaluate the 1,1-diphenyl-2-picrylhydrazyl (DPPH) radical scavenging activity of a methanol extract of these flowers at a concentration of 40 μg/mL. The values obtained ranged from 3.4% to 91.3% (Figure 1a). O. fragrans exhibited the strongest activity (91.3%), but a little less activity than green tea (93.4%), which has been proven to exhibit strong antioxidative activity [1,2]. Similarly, the total phenolic content of O. fragrans (291.3 mg/g extract) was determined to be a little less than that of green tea (325.9 mg/g extract), but it was the highest among the seven flowers tested (Figure 1b). Therefore, O. fragrans became the candidate for a constitution and pharmacological investigation.

*Osmanthus* is a genus of approximately 30 species of flowering plants of the family Oleaceae, mostly native to warm temperature regions of Asia ranging from the east of the Himalaya through southern China to Taiwan and southern Japan. The species *Osmanthus fragrans* Lour. is an evergreen, polygamous or dioecious shrub or small tree, which grows to 3–12 m tall. Its flowers are white, pale yellow, golden yellow, or orange yellow with a four-lobed corolla, and have a strong fragrance [3]. It is used not only as an ornamental plant, but also as an additive in food, tea, and other beverages because of its strong fragrance. Most components in the essential oil of *O. fragrans* have been identified by GC-MS [4,5]. Additionally, the flower of *O. fragrans* has been demonstrated to exhibit antioxidative activity [6], to inhibit NO production [7], to have a neuroprotective effect [8], and to inhibit melanogenesis [9,10]. It can be utilized as a herbal drug against toothache, as a gargle [11], or in cosmetic therapeutics [12]. However, the constituents responsible for the antioxidative effect are unknown. This study describes the isolation, structural elucidation of the purified compounds, and evaluates their antioxidative activity against DPPH radical and hydrogen peroxide (H_2_O_2_).

## 2. Results and Discussion

### 2.1. Antioxidative Activity and Total Phenolic Content of Extracts

The antioxidative activity of the crude methanol extract was determined to be 91.3% at 40 μg/mL (IC_50_ = 12.8 μg/mL) in the scavenging of DPPH radical and 72.4% at 24 μg/mL (IC_50_ = 16.6 μg/mL) in the scavenging of H_2_O_2_ (Table 1). The total phenolic content was found to be 291.3 mg/g extract. Common natural antioxidants are phenolics, ascorbic acid, tocopherols, and carotenes. Ascorbic acid, tocopherols, and carotenes contents were not detected herein by the 2,6-dichloroindophenol (DIP) method or HPLC analysis [13]. Accordingly, the phenolic compounds were the dominant antioxidants in the methanol extract of *O. fragrans*. 

The crude methanol extract of *O. fragrans* was suspended in H_2_O. The aqueous solution was partitioned with CHCl_3_ to yield the CHCl_3_ sub-extract. It exhibited strong antioxidative activity (87.4% at 40 μg/mL, IC_50_ = 20.9 μg/mL). The phenolic components associated with the antioxidative activity of the CHCl_3_ sub-extract were thus investigated.

**Table 1:** DPPH radical and H_2_O_2_ scavenging activities of methanol and CHCl_3_ sub-extract. nd: not determined.

		DPPH scavenging effects	H_2_O_2_ scavenging effects
		IC_50_ (μg/mL)	IC_50_ (μg/mL)
MeOH extract		12.8	16.6
CHCl_3_ sub-extract		20.9	nd

nd: not determined.

### 2.2. Isolation and Structural Characterization of Phenolics

**Figure 2:**
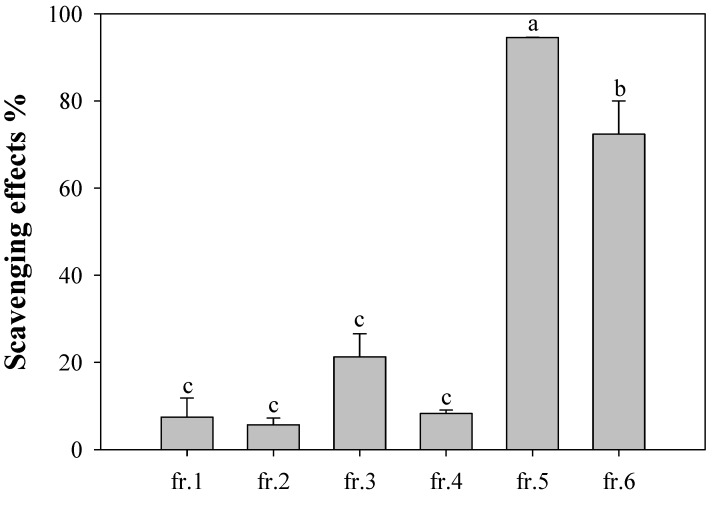
Scavenging effect of fractions 1–6 from chloroform sub-extract of the flower of *O. fragrans*. The concentration of each solution was 40 μg/mL. ^a–c^ Values in each column with different letters are significantly different (*p* *<* 0.05). Each value is mean ± standard derivation of three replicate analysis.

**Figure 3:**
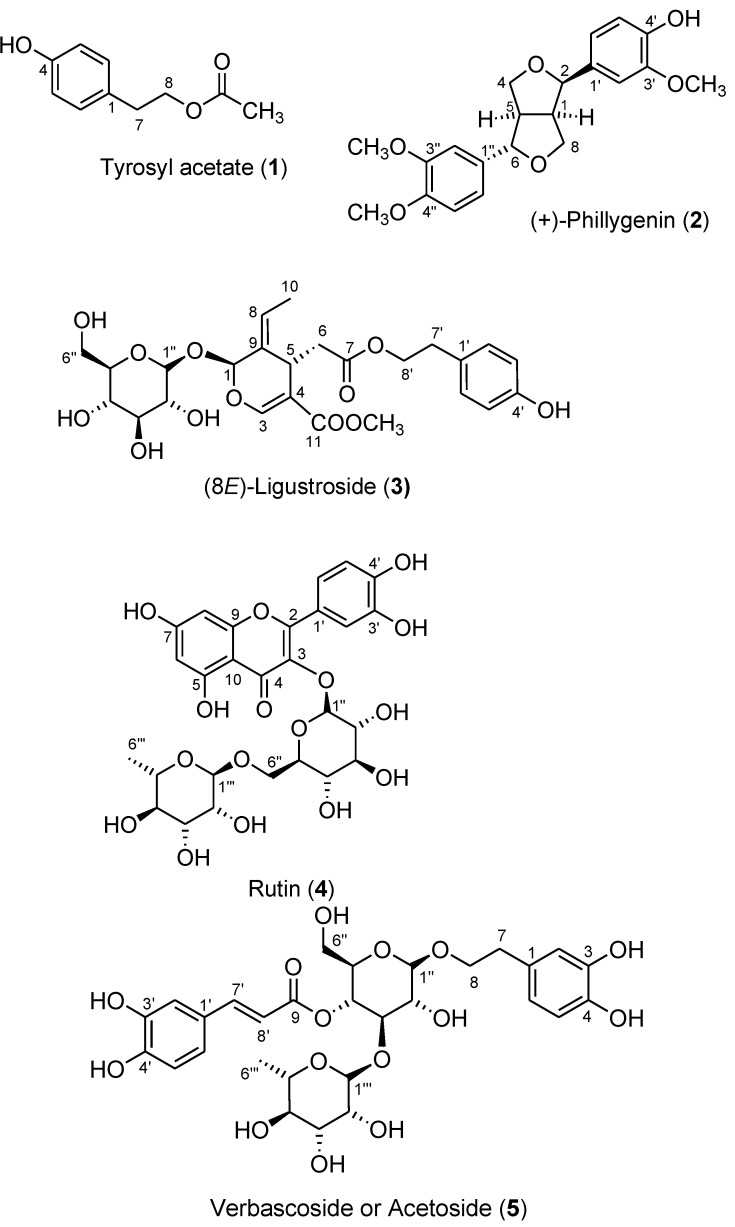
Structures of compounds **1**–**5** isolated from the CHCl_3_ sub-extract of *Osmanthus fragrans*.

Successive silica gel column chromatographic separations of the obtained CHCl_3_ sub-extract yielded six fractions. Figure 2 presents the antioxidative activities of these fractions at 40 μg/mL; fractions 5 and 6 exhibited high antioxidativity and fraction 3 exhibited moderate activity. Five phenolic compounds—tyrosyl acetate [*p*-(hydroxyphenyl)ethyl acetate, **1**], (+)-phillygenin (**2**), (8*E*)-ligustroside (**3**), rutin (quercetin-3-rutinoside, **4**), verbascoside (acteoside, **5**)—were isolated from fractions 2−6, respectively, by repeated chromatography (Figure 3). The structures of these compounds were established using UV, IR, ^1^H- and ^13^C-NMR, 2D-NMR (COSY, HMQC, HMBC, and NOESY), MS and specific rotation data. All compounds, except **2**, were isolated from this plant for the first time.

From fraction 2, a white amorphous powder compound **1** was isolated. The pseudo molecular ion at an *m/z* of 181 [M+H]^+^ in the FABMS had the molecular formula C_10_H_12_O_3_. The ^1^H-NMR spectrum presented signals at δ 6.77 (2H, d, *J* = 8.0 Hz) and 7.07 (2H, d, *J* = 8.0 Hz) typical of a *para*-disubstituted benzene. A hydroxyl substituent on the ring was responsible for the broad signal at δ 5.29.

The other substituent was assumed to be an acetoxyethyl group associated with signals at δ 2.04 (3H, s), 2.86 (2H, t, *J* = 7.0 Hz), 4.24 (2H, t, *J* = 7.0 Hz). Therefore, compound **1** was determined to be *p*-(hydroxyphenyl)ethyl acetate, which is also called tyrosyl acetate [14].

From fraction 3, compound **2** was obtained as a white amorphous powder. The FABMS revealed I to be a molecule with a formula of C_21_H_24_O_6_, based on the presence of a pseudo molecular ion at an *m/z* of 373 [M*+*H]^+^. The UV and IR spectra suggested the presence of an aromatic ring. In the ^1^H-NMR and COSY spectra, aromatic signals at δ 6.78 (1H), 6.83 (1H), 6.91 (2H) and 6.99 (2H) together with three methoxyl signals at δ 3.78, 3.80, and 3.83 and a hydroxyl signal at δ 7.58 revealed the presence of a 4'-hydroxy-3'-methoxyphenyl ring and a 3",4"-dimethoxyphenyl ring. The eight aliphatic signals at 2.89 (1H), 3.18 (1H), 3.39 (1H), 3.76 (2H) 4.10 (1H), 4.35 (1H), 4.83 (1H) represent two sets of mutually coupled CH_2_-CH-CH fragments that are linked by two oxygen atoms to form an unsymmetrical furofuran lignan. The *cis*-fused furofuran ring system was revealed by the strong NOE correlation between H-1 and H-5. The NOE correlation between H-1 and H-2, the absence of an NOE correlation between H-5 and H-6 and the positive specific rotation ([α]_D_ = +88°) suggest that the absolute structure of **2** is that of (+)-phillygenin [15].

From fraction 4, compound **3** was obtained as a white amorphous powder. The UV and IR spectra revealed hydroxyl, carboxyl, and aromatic functionalities. The molecular formula C_25_H_32_O_12_ was verified by FABMS at an *m/z* 547 for the pseudo molecular [M*+*Na]^+^ ion. Analyzing the ^1^H, ^13^C-NMR, COSY, HMQC and HMBC spectra revealed a set of glucose signals (anomeric proton at 4.83 d, *J* = 7.7 Hz), aglycone signals that were associated with a (*p*-hydroxyphenyl)ethyl(tyrosyl) acetate moiety at δ 2.80 (2H, H-7'), 4.08 and 4.20 (each 1H, H-8'), 6.76 (2H, H-3' and H-5'), 7.07 (2H, H-2' and H-6'), and 8.35 (1H, 4'-OH), an ethylidene signals at δ 1.66 (3H, H-10) and 6.02 (1H, H-8), and a signal at δ 3.67 associated with a methoxycarbonyl substituent that were attached to a dihydropyran ring. The HMBC correlations of H-1" with C-1, suggesting the attachment of a glucose to C-1; H-8 with both C-1 and C-5, suggesting ethylidene on C-9; H-6 with both C-9 and C-4, suggesting the attachment of *p*-tyrosyl acetate to C-5, and both H-5 and H-3 with C-11, suggesting the attachment of methoxycarbonyl to C-4. Spectroscopic details were consistent with those reported elsewhere for secoiridoid ligustroside. The NOE correlation between H-7 and H-3 verified the *E* configuration. Therefore, compound **3** was determined to be (8*E*)-ligustroside [16].

From fraction 5, a yellow needle-like crystalline substance with molecular formula C_27_H_30_O_16_ was identified based on the pseudomolecular ion at an *m/z* of 611 [M*+*H]^+^ in FABMS, and was obtained as compound **4**. Two sugar units, glucosyl (anomeric proton signal at δ 5.10 d, *J* = 7.2 Hz) and rhamnosyl (anomeric proton signal at δ 4.53 br s and a methyl signal at δ 1.13 d, *J* = 6.0 Hz), were identified from the ^1^H- and ^13^C-NMR and HMQC spectra. The remaining aglycone signals in the ^1^H-NMR spectrum, associated with an ABX system at δ 6.87 (d, *J* = 8.3 Hz, H-5'), 7.62 (dd, *J* = 8.3, 1.9 Hz, H-6'), and 7.67 (1H, d, *J* = 1.9 Hz, H-2') and an AB system at δ 6.18 (d, *J* = 1.5 Hz, H-6) and 6.36 (d, *J* = 1.5 Hz, H-8), are attributed to a quercetin moiety. Hence, the structure of compound **4** was established as quercetin 3-*O*-rhamnosyl(1→6) glucoside. This compound was called rutin. The full ^1^H and ^13^C assignments of this flavonoid were confirmed by 2D-COSY, HMQC, HMBC, and NOESY experiments [17].

From fraction 6, a compound **5** was isolated as a white amorphous powder. Its molecular weight was 624, as determined by FABMS, consistent with the molecular formula C_29_H_36_O_15_. The UV and IR spectra showed the presence of hydroxyl and conjugated carbonyl functional groups. Like rutin (**4**), two sugars, a glucose and a rhamnose, were revealed by the anomeric signals at δ 4.38 (d, *J* = 7.8 Hz) and 5.19 (br s) and a methyl signal at δ 1.10 (d, *J* = 6.0 Hz). Furthermore, a caffeoyl moiety was revealed by 3,4-dihydroxyphenyl signals at δ 6.78 (d, *J* = 8.1 Hz), 6.96 (d, *J* = 8.1 Hz), and 7.06 (s) and *trans* double bond signals at 6.28 and 7.60 (d, *J* = 15.9 Hz). A (3,4-dihydroxyphenyl)ethyl, called hydroxytyrosyl, moiety was identified from the aromatic signals at δ 6.57 (d, *J* = 7.9 Hz, H-6), 6.68 (d, *J* = 7.9 Hz, H-5), 6.70 (s, H-2) and from the aliphatic signals at δ 2.80 (H-7) and 3.72 and 4.05 (H-8). The key HMBC correlations of glucosyl H-1" with phenylethyl C-8; glucosyl H-4" with caffeoyl C-9', glucosyl H-3" with rhamnosyl C-1"' revealed that **5** had the verbascoside (acteoside) structure of 1-*O*-(3,4-dihydroxyphenyl)ethyl 4-*O*-caffeoyl-3-*O*-rhamnosyl(1→3)glucose. The spectral data of **5** agreed closely with those reported in the literature [18].

If we focus on the aromatic rings, the structures of phenolic compounds **1**–**5** differ mainly in the number and position of hydroxyl or methoxyl groups that are attached to the phenyl ring: tyrosyl acetate (**1**) has a tyrosyl group with only a *p*-phenolic hydroxyl group; (+)-phillygenin (**2**) contains two methylated catechol moieties—one with a methoxyl group and a free hydroxyl group, the other with two methoxyls; (8*E*)-ligustroside (**3**) has a tyrosyl unit such as **1**; rutin (**4**) is a flavonoid with two hydroxyl groups in the B ring and a rutinose that is attached to 3-OH; verbascoside (**5**) has four phenolic hydroxyl groups between both hydroxytyrosyl and caffeoyl units.

### 2.3. Evaluation of Antioxidative Activity of Isolated Phenolics

Phenolic compounds are believed to be scavengers of free radicals. Their antioxidative activity depends on their chemical structure; specifically, it depends on their ability to donate hydrogen or electron from the aromatic structure. Different antioxidants respond differently in different measurement methods.

#### 2.3.1. DPPH Radical Scavenging Activity

**Figure 4:**
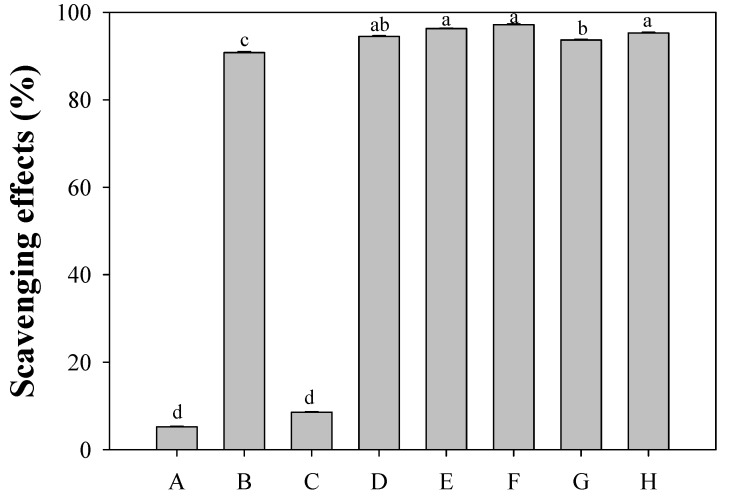
Scavenging effect of isolated compounds from chloroform sub-extract of the flower of *O. fragrans* on DPPH radicals. (A) *p*-tyrosyl acetate (**1**); (B) phillygenin (**2**); (C) ligustroside (**3**); (D) rutin (**4**); (E) verbascoside (**5**); (F) quercetin; (G) gallic acid; (H) Trolox. The concentration of each solution was 40 μg/mL. ^a^^–d^ Values in each column with different letters are significantly different (*p* < 0.05). Each value is mean ± standard derivation of three replicate analysis.

The free radical scavenging activities of pure phenolics **1**–**5** were firstly determined by measuring their ability to transfer hydrogen to a stable DPPH radical. The antioxidants quercetin, gallic acid, and Trolox as a reference were used (Figure 4). At a fixed concentration of 40 μg/mL, the inhibition decreased in the order quercetin (97.2%) > verbascoside (**5**) (96.3%) ≥ Trolox (95.3%) ≥ rutin (**4**) (94.5%) > gallic acid (93.7%) > (+)-phillygenin (**2**) (90.8%) > (8*E*)-ligustroside (**3**) (8.6%) > tyrosyl acetate (**1**) (5.2%) (Figure 4). The last two phenolics, tyrosyl acetate (**1**) and (8*E*)-ligustroside (**3**), containing a tyrosyl unit, contributed almost nothing to the anti-radical scavenging activity [19,20]. In further analysis, the IC_50_ values of verbascoside (**5**), rutin (**4**), (+)-phillygenin (**2**) were calculated to be 6.2, 10.3, and 19.1 μM, respectively, based on molar concentration (Table 2). Phenolic compounds **5** and **4** exhibited much greater antioxidative activity than gallic acid and Trolox (IC_50_, 27.0 and 19.6 μM), while **2** had a similar activity to that of Trolox but a stronger activity than that of gallic acid. Quercetin, the aglycone of rutin, exhibited greater DPPH scavenging activity than rutin (**4**), indicating blocking of the 3-OH in the flavonoid by a glycoside reduced the antioxidative activity [21].

**Figure 5:**
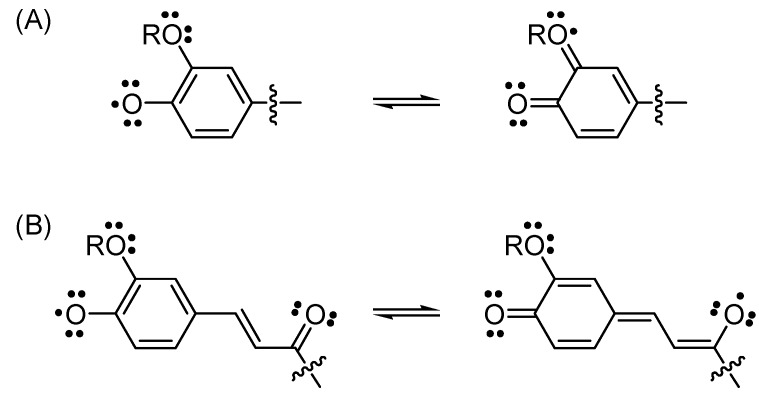
Resonance structures of *ortho*-oxygenated *p*-hydroxyphenyl radical (**A**) and *p*-hydroxycinnamoyl radical (**B**).

When a comparison was made among **1**–**5**, compounds **5**, **4**, and **2** exhibited greater antioxidative activity than compounds **3** and **1**, because **5** and **4** have an additional hydroxyl group and **2** has a methoxyl group *ortho* to the *p*-hydroxylphenyl moiety. Compounds **5** and **4** were significantly better radical scavengers than compound **2**, suggesting that the hydroxyl group had a stronger effect on the antioxidative activity than does the methoxyl group. Moreover, a C=C double bond next to a carbonyl group that was conjugated with a catechol in verbascoside (**5**) and a catechol B-ring in rutin (**4**) further strengthened antioxidative activity. The electron-donating oxygenated group and C=C−C=O moiety may have participated in stabilizing the *ortho*-oxygenated *p*-hydroxyphenyl radical and *p*-hydroxycinnamoyl radical by delocalization of the unpaired electron toward the *ortho*-oxygen atom (Figure 5A) or toward the ketone functionality (Figure 5B). These observations are consistent with the literature [13,22,23,24,25,26].

#### 2.3.2. Hydrogen Peroxide Scavenging Activity

Biological systems can generate H_2_O_2_, which is known to be a non-radical oxidant that is not very reactive. It is sometimes toxic and induces cell death because it can form the hydroxyl radical HO·, which is the most potent and reactive species [27]. Accordingly, the effective scavenging of H_2_O_2_ can prevent the oxidative damage of cells.

In our preliminary screening, tyrosyl acetate (**1**) and (8*E*)-ligustroside (**3**) did not exhibit any significant H_2_O_2_ scavenging activity. Owing to the structural difference, the activity was adopted as molar concentration. The IC_50_ of the test compounds (expressed in μM) revealed that the activity followed the order gallic acid > (+)-phillygenin (**2**) > verbascoside (**5**) > quercetin > Trolox > rutin (**4**) (Table 2). These results demonstrate that the compounds that contain having catechol or a methylated-catechol group—which were **2**, **4**, and **5**—exhibited greater H_2_O_2_ activity than compounds that contained one phenolic hydroxyl group—which were **1** and **3**. This finding is consistent with the study of Ozyurek *et al.* [28]. Among the three isolated phenolics, **2**, **4**, and **5**, the strongest H_2_O_2_ scavenger was (+)-phillygenin (**2**) while the weakest one was rutin (**4**). Even though all three compounds were less active than the reference gallic acid, compounds **2** and **5** exhibited stronger H_2_O_2_ scavenging activity than quercetin and Trolox. Rutin (**4**) exhibited lower activity than its aglycone, quercetin. The strong activity of (+)-phillygenin (**2**) suggested that the methoxyl groups on the phenyl ring were more effective than the free hydroxyl group in scavenging H_2_O_2_.

**Table 2:** DPPH radical and H_2_O_2_ scavenging activities of isolated compounds, **2**, **4**, and **5**.

		DPPH scavenging effects		H_2_O_2_ scavenging effects
		IC_50_ (μM)	IC_50_ (μM)	
(+)-phillygenin (**2**)		19.1		10.5
Rutin (**4**)		10.3		23.4
Verbascoside (**5**)		6.2		13.4
Quercetin		7.3		15.9
Gallic acid		27.0		8.2
Trolox		19.6		17.2

## 3. Experimental

### 3.1. General

Optical rotations were measured on a Jasco DIP-370 digital polarimeter. UV/VIS absorbances and spectra were recorded on an Agilent 8453, Hitachi U-2001, or Jasco V-630 bio spectrophotometer. IR spectra were recorded on a Nicolet Magna FT-IR spectrophotometer. ^1^H and ^13^C-NMR, DEPT, COSY, HMQC, HMBC, and NOESY spectra were recorded on Bruker Avance-300 FT-NMR spectrometers; all chemical shifts were given in ppm from tetramethylsilane as an internal standard. Mass spectra were obtained on VG 70-250S spectrometer by a direct inlet system. Ethyl acetate (EtOAc), methanol (MeOH), hexane, chloroform (CHCl_3_), 2,2-diphenyl-1-picrylhydrazyl (DPPH), 30 wt.% H_2_O_2_, Na_2_HPO_4_, KH_2_PO_4_, NaCl, phenol red, horseradish peroxidase (type II), quercetin, gallic acid, 6-hydroxy-2,5,7,8-tetramethylchromane-2-carboxylic acid (Trolox), Na_2_CO_3_, and Folin-ciocalteau reagent were purchased from Sigma Chemicals. (St. Louis, MO, USA). Silica gel (70–230 mesh) and TLC plates were purchased from Merck Chemicals (Darmstadt, Germany).

### 3.2. Plant Material

The dried flowers of *Gomphrena globosa*, *Helichrysum bracteatum*, *Chrysanthemum morifolium*, *Momordica grosvenori*, *Chrysanthemum indicum*, and *Osmanthusfragrans* were purchased from a traditional market at Guilin, Guangxi Province, China, in 2007. The dried flower of *Nelumbo nucifera* and green tea were purchased from a market at Liujia, Tainan County, Taiwan, in 2008. Voucher specimen of *Osmanthus fragrans* (HCY080801) has been deposited at the herbarium of the Department of Food Nutrition, Chung Hwa University of Medical Technology, Tainan, Taiwan.

### 3.3. Extraction of Seven Flowers and Green Tea

One gram of the above seven flowers, *G. globosa*, *H. bracteatum*, *C. morifolium*, *M. grosvenori*, *C. indicum*, *N. nucifera*, and *O. fragrans* together with green tea (for comparison) was individually extracted with MeOH (30 mL) for 12 h. The filtration was concentrated under reduced pressure to give methanol extract for subsequent antioxidative activity assay. 

### 3.4. Extraction and Isolation of Osmanthus fragrans

The dried flower of *O. fragrans* (2.8 kg) were extracted with MeOH (3 × 5 L) under reflux for 8 h. The combined extracts were concentrated under reduced pressure to give dark brown syrup (138 g) and some yellow crystals (5.2 g). The syrup was then suspended in H_2_O and partitioned with CHCl_3_. The CHCl_3_ soluble sub-extract (90 g) was chromatographed on a silica gel column eluting with pure hexane, hexane/EtOAc (3:1, 1:1, and 1:3), pure EtOAc, and finally pure MeOH, to yield six fractions. After repeated chromatography on a silica gel column, five phenolic compounds, tyrosyl acetate (**1**, 6.3 mg, 0.00023%), (+)-phillygenin (**2**, 18.5 mg, 0.00066%), (8*E*)-ligustroside (**3**, 71.1 mg, 0.0025%), rutin (**4**), and verbascoside (**5**), were obtained from fractions 2, 3, 4, 5, and 6, respectively. The yellow crystals were subjected to column chromatography on a silica gel column eluting with pure EtOAc and pure MeOH to give rutin (**4**) and verbascoside (**5**), respectively. The combined rutin (**4**) weighted 1.26 g (0.045%) and the verbascoside (**5**) 2.85 g (0.12%).

*Tyrosyl acetate* (**1**). C_10_H_12_O_3_, white amorphous powder. UV (MeOH) *λ_max_* 224, 278, 309 nm; IR (KBr) *ν_max_* 3390, 1714, 1614, 1518 cm^−1^; ^1^H-NMR (CDCl_3_) *δ* 2.04 (3H, s, CH_3_), 2.86 (2H, t, *J* = 7.0 Hz, H-7), 4.24 (2H, t, *J* = 7.0 Hz, H-8), 5.29 (1H, br s, 4-OH), 6.77 (2H, d, *J* = 8.0 Hz, H-3 and H-5), 7.07 (2H, d, *J* = 8.0 Hz, H-2 and H-6); FABMS *m/z* (rel. int.) 181 (22, [M*+*H]^+^).

*(+)-Phillygenin* (**2**). C_21_H_24_O_6_, white amorphous powder. [α]_D_ +88° (c 0.07, CHCl_3_); UV (MeOH) *λ_max_* 229, 280 nm; IR (KBr) *ν_max_* 3400, 1614, 1517 cm^−1^; ^1^H-NMR (acetone-*d*_6_) *δ*2.89 (1H, m, H-1), 3.18 (1H, t, *J* = 8.7 Hz, H-4a), 3.39 (1H, m, H-5), 3.76 (2H, m, H-4b and H-8a), 3.78 (3H, s, 4"-OCH_3_), 3.80 (3H, s, 3"-OCH_3_), 3.83 (3H, s, 3'-OCH_3_), 4.10 (1H, d, *J* = 9.2 Hz, H-8b), 4.35 (1H, d, *J* = 7.0 Hz, H-2), 4.83 (1H, d, *J* = 6.0 Hz, H-6), 6.78 (1H, d, *J* = 8.0 Hz, H-5'), 6.83 (1H, d, *J* = 8.0 Hz, H-6'), 6.91 (2H, br s, H-5" and H-6"), 6.99 (2H, m, H-2' and H-2"), 7.58 (1H, br s, 4'-OH); ^13^C-NMR (acetone-d_6_) *δ* 50.8 (C-5), 55.6 (C-1), 56.1 (3'-, 3"-, and 4"-OCH_3_), 70.0 (C-4), 71.5 (C-8), 82.5 (C-6), 88.5 (C-2), 110.5 (C-2"), 110.6 (C-2'), 112.4 (C-5"), 115.5 (C-5'), 118.6 (C-6"), 119.6 (C-6'), 132.5 (C-1"), 134.2 (C-1'), 146.9 (C-4'), 148.3 (C-3'), 149.2 (C-4"), 150.1 (C-3"); FABMS *m/z* (rel. int.) 373 (30, [M*+*H]^+^).

*(8E)-Ligustroside* (**3**). C_25_H_32_O_12_, white amorphous powder. [α]_D_ −172 (c 1.14, acetone); UV (MeOH) *λ_max_* 227, 278 nm; IR (KBr) *ν_max_* 3417, 1714, 1633, 1518 cm^−1^; ^1^H-NMR (acetone-*d*_6_) *δ*1.66 (3H, d, *J* = 6.9 Hz, H-10), 2.40 (1H, dd, *J* = 14.3, 9.5 Hz, H-6a), 2.71 (1H, dd, *J* = 14.3, 4.1 Hz, H-6b), 2.80 (2H, t, *J* = 7.0 Hz, H-7'), 3.37 (3H, m, H-2", H-4", and H-5"), 3.49 (1H, m, H-3"), 3.65 (1H, m, H-6"a), 3.67 (3H, s, OCH_3_), 3.83 (1H, m, H-6"b), 3.95 (1H, dd, *J* = 9.5, 4.1 Hz, H-5), 4.08 (1H, m, H-8'a), 4.20 (1H, m, H-8'b), 4.83 (1H, d, *J* = 7.7 Hz, H-1"), 5.92 (1H, s, H-1), 6.02 (1H, q, *J* = 6.9 Hz, H-8), 6.76 (2H, d, *J* = 8.4 Hz, H-3' and H-5'), 7.07 (2H, d, *J* = 8.4 Hz, H-2' and H-6'), 7.47 (1H, s, H-3), 8.35 (1H, br s, 4'-OH); ^13^C-NMR (acetone-*d*_6_) *δ* 13.5 (C-10), 31.4 (C-5), 34.7 (C-7'), 40.7 (C-6), 51.5 (OCH_3_), 62.8 (C-6"), 66.1 (C-8'), 71.4 (C-4"), 74.5 (C-2"), 77.7 (C-3"), 77.9 (C-5"), 94.5 (C-1), 100.5 (C-1"), 109.1 (C-4), 116.1 (C-3' and C-5'), 124.1 (C-8), 129.5 (C-1'), 130.3 (C-9), 130.7 (C-2' and C-6'), 154.2 (C-3), 156.9 (C-4'), 167.2 (C-11), 171.7 (C-7); FABMS *m/z* (rel. int.) 547 (100, [M*+*Na]^+^).

*Rutin* (**4**). C_27_H_30_O_16_, yellow needle-like crystal. [α]_D_ −25° (c 0.15, CH_3_OH); UV (MeOH) *λ_max_* 208, 251, 365, 398 nm; IR (KBr) *ν_max_* 3322, 1656, 1603, 1564, 1505 cm^−1^; ^1^H-NMR (CD_3_OD) *δ*1.13 (3H, d, *J* = 6.0 Hz, H-6'"), 3.29 (1H, t, *J* = 9.4 Hz, H-4'"), 3.32 (2H, m, H-4" and H-5"), 3.39 (1H, dd, *J* = 9.9, 6.6 Hz, H-6"a), 3.43 (2H, m, H-3" and H-5'"), 3.47 (1H, m, H-2"), 3.54 (1H, t, *J* = 9.4 Hz, H-3'"), 3.66 (1H, br s, H-2'"), 3.81 (1H, br d. *J* = 9.9 Hz, H-6"b), 4.53 (1H, br s, H-1'"), 5.10 (1H, d, *J* = 7.2 Hz, H-1"), 6.18 (1H, d, *J* = 1.5 Hz, H-6), 6.36 (1H, d, *J* = 1.5 Hz, H-8), 6.87 (1H, d, *J* = 8.3 Hz, H-5'), 7.62 (1H, dd, *J* = 8.3, 1.9 Hz, H-6'), 7.67 (1H, d, *J* = 1.9 Hz, H-2'); ^13^C-NMR (CD_3_OD) *δ* 17.9 (C-6'"), 68.5 (C-6"), 69.7 (C-5'"), 71.3 (C-4"), 72.0 (C-2'"), 72.2 (C-3'"), 73.9 (C-4'"), 75.7 (C-2"), 77.1 (C-5"), 78.1 (C-3"), 94.9 (C-8), 99.9 (C-6), 102.4 (C-1'"), 104.8 (C-1"), 105.6 (C-10), 116.0 (C-5'), 117.7 (C-2'), 123.1 (C-1'), 123.6 (C-6'), 135.6 (C-3), 145.7 (C-3'), 149.7 (C-4'), 158.4 (C-9), 159.3 (C-2), 162.8 (C-5), 165.9 (C-7), 179.3 (C-4); FABMS *m/z* (rel. int.) 611 (10, [M*+*H]^+^).

*Verbascoside* (**5**). C_29_H_36_O_15_, white amorphous powder. [α]_D_ −89° (c 0.68, CH_3_OH); UV (MeOH) *λ_max_* 210, 258, 365 nm; IR (KBr) *ν_max_* 3403, 1697, 1603, 1525 cm^−1^; ^1^H-NMR (CD_3_OD) *δ*1.10 (3H, d, *J* = 6.0 Hz, H-6'"), 2.80 (2H, t, *J* = 7.0 Hz, H-7), 3.31 (1H, m, H-4'"), 3.40 (1H, dd, *J* = 9.0, 7.8 Hz, H-2"), 3.5–3.7 (5H, m, H-3'", H-5", H-5'", and H-6"), 3.72 (1H, m, H-8a), 3.82 (1H, t, *J* = 9.0 Hz, H-3"), 3.93 (1H, br s, H-2'"), 4.05 (1H, m, H-8b), 4.38 (1H, d, *J* = 7.8 Hz, H-1"), 4.91 (1H, t, *J* = 9.0 Hz, H-4"), 5.19 (1H, br s, H-1'"), 6.28 (1H, d, *J* = 15.9 Hz, H-8'), 6.57 (1H, d, *J* = 7.9 Hz, H-6), 6.68 (1H, d, *J* = 7.9 Hz, H-5), 6.70 (1H, s, H-2), 6.78 (1H, d, *J* = 8.1 Hz, H-5'), 6.96 (1H, d, *J* = 8.1 Hz, H-6'), 7.06 (1H, s, H-2'), 7.60 (1H, d, *J* = 15.9 Hz, H-7'); ^13^C-NMR (CD_3_OD) *δ* 18.4 (C-6'"), 36.5 (C-7), 62.3 (C-6"), 70.4 (C-4"), 70.6 (C-5'"), 72.0 (C-3'"), 72.2 (C-8), 72.3 (C-2'"), 73.8 (C-4'"), 76.0 (C-2"), 76.2 (C-5"), 81.6 (C-3"), 103.0 (C-1'"), 104.2 (C-1"), 114.6 (C-8'), 115.2 (C-2'), 116.3 (C-5'), 116.5 (C-5), 117.1 (C-2), 121.3 (C-6), 123.2 (C-6'), 127.6 (C-1'), 131.5 (C-1), 144.6 (C-4), 146.1 (C-3), 146.9 (C-3'), 148.0 (C-7')149.9 (C-4'), 168.3 (C-9'); FABMS *m/z* (rel. int.) 625 (100, [M*+*H]^+^).

### 3.5. Evaluation of DPPH Radical Scavenging Activity

#### 3.5.1. DPPH Radical Scavenging Assay

This DPPH assay was investigated by the modified method of Shimada *et al.* [29]. MeOH (3.8 mL), sample solution in methanol (0.2 mL, 1 mg/mL), and 1 mM DPPH solution (1.0 mL) were well mixed and left to stand in the dark at room temperature for 30 min. The final concentration of the sample is 40 μg/mL. The absorbance at 517 nm was measured Sample in methanol was used as blank, while DPPH radical in methanol solution was used as a control. Then, the DPPH radical scavenging activity was calculated according to the following equation:

% of DPPH radical scavenging activity = [1 − (A_sample_ − A_blank_)/A_control_] × 100

The concentration providing 50% inhibition (IC_50_) was calculated from the plot of inhibition percentage against sample concentration by linear regression analysis. 

#### 3.5.2. Hydrogen Peroxide Scavenging Assay

Hydrogen peroxide (H_2_O_2_) scavenging activity was measured by the modified method of Sroka and Cisowski [30]. Sample solution in water (100 μL, 1 mg/mL), 0.002% H_2_O_2_ (100 μL) and 0.1 M phosphate buffer (Na_2_HPO_4_:KH_2_PO_4_, pH 7.4, 0.8 mL) containing 100 mM NaCl were mixed thoroughly. Then 0.3 mg/mL phenol red (1 mL) with horseradish peroxidase (0.2 mg/mL) 0.1 M phosphate buffer was added. After 15 min at room temperature, a solution of 1 M NaOH (50 μL) was added and the absorption at 610 nm was measured immediately. Water solution without sample was used as a control. Then, the H_2_O_2_ scavenging activity was calculated according to the following equation:

% of H_2_O_2_ scavenging activity = [(A_control_ – A_sample_)/Acontrol] × 100

The concentration providing 50% inhibition (IC_50_) was calculated from the plot of inhibition percentage against sample concentration by linear regression analysis. 

### 3.6. Determination of Total Phenolic Content

According to the method described by Yen and Hung [31], sample solution in methanol (0.1 mL, 1 mg/mL) was well mixed with 2% Na_2_CO_3_ (2 mL). After an interval of 3 min, 50% Folin-Ciocalteau reagent (0.1 mL) was added. The mixture was allowed to stand at room temperature for 30 min with intermittent mixing. The absorbance at 750 nm was recorded. A standard curve using gallic acid was prepared. The total phenolic content was expressed as gallic acid equivatlents (mg of GAE per g of sample).

### 3.7. Statistical Analysis

Each value was expressed as a mean ± SD. At least triplicate experiments were conducted. We analyzed the variance using ANOVA followed by Duncan’s multiple range test to determine which means were significantly different from each other or the control. In all cases, a *p* value of <0.05 was used to determine the significance. 

## 4. Conclusions

The methanol extracts of green tea and seven flowers, *G. globosa*, *H. bracteatum*, *C. morifolium*, *M. grosvenori*, *C. indicum*, *N. nucifera*, and *O. fragrans* were subjected to a DPPH radical scavenging activity test. Of these, *O. fragrans* exhibited strong antioxidative activity that was only a little less than that of green tea. The extract of *O. fragrans* was suspended in water and extracted with CHCl_3_. From the CHCl_3_ sub-extract of *O. fragrans*, five phenolic compounds, namely tyrosyl acetate (**1**), (+)-phillygenin (**2**), (8*E*)-ligustroside (**3**), rutin (**4**), and verbascoside (**5**), were isolated. (+)-Phillygenin (**2**), rutin (**4**), and verbascoside (**5**) with a catechol-like or dihydroxycinnamoyl-like structural feature exhibited strong scavenging activity toward DPPH radical and H_2_O_2_. The compounds tyrosyl acetate (**1**) and (8*E*)-ligustroside (**3**), with a *p*-hydroxyphenyl moiety, exhibited very low activity. Verbascoside (**5**) was obtained in a high yield and exhibited a high antiradical activity; phillygenin (**2**) was a strong H_2_O_2_ quencher. Therefore, phenolic compounds **2**, **4**, and **5** in *O. fragrans* appeared to be significant antioxidants. The water portion may contain other unknown antioxidative compounds. Further work must be undertaken to determine this. 
